# A Transgenic Transcription Factor (TaDREB3) in Barley Affects the Expression of MicroRNAs and Other Small Non-Coding RNAs

**DOI:** 10.1371/journal.pone.0042030

**Published:** 2012-08-01

**Authors:** Michael Hackenberg, Bu-Jun Shi, Perry Gustafson, Peter Langridge

**Affiliations:** 1 Department of Genetics, University of Granada, Granada, Granada, Spain; 2 Australian Centre for Plant Functional Genomics, University of Adelaide, Adelaide, South Australia, Australia; 3 Division of Plant Sciences, University of Missouri, Columbia, Missouri, United States of America; TGen, United States of America

## Abstract

Transcription factors (TFs), microRNAs (miRNAs), small interfering RNAs (siRNAs) and other functional non-coding small RNAs (sRNAs) are important gene regulators. Comparison of sRNA expression profiles between transgenic barley over-expressing a drought tolerant TF (*TaDREB3*) and non-transgenic control barley revealed many group-specific sRNAs. In addition, 42% of the shared sRNAs were differentially expressed between the two groups (|log_2_| >1). Furthermore, *TaDREB3*-derived sRNAs were only detected in transgenic barley despite the existence of homologous genes in non-transgenic barley. These results demonstrate that the TF strongly affects the expression of sRNAs and siRNAs could in turn affect the TF stability. The TF also affects size distribution and abundance of sRNAs including miRNAs. About half of the sRNAs in each group were derived from chloroplast. A sRNA derived from tRNA-His(GUG) encoded by the chloroplast genome is the most abundant sRNA, accounting for 42.2% of the total sRNAs in transgenic barley and 28.9% in non-transgenic barley. This sRNA, which targets a gene (TC245676) involved in biological processes, was only present in barley leaves but not roots. 124 and 136 miRNAs were detected in transgenic and non-transgenic barley, respectively. miR156 was the most abundant miRNA and up-regulated in transgenic barley, while miR168 was the most abundant miRNA and up-regulated in non-transgenic barley. Eight out of 20 predicted novel miRNAs were differentially expressed between the two groups. All the predicted novel miRNA targets were validated using a degradome library. Our data provide an insight into the effect of TF on the expression of sRNAs in barley.

## Introduction

Transcription factors (TFs) are important regulators of gene transcription in organisms and have been classified into a number of families based on their DNA-binding domain sequences and structures. Plant-specific APETALA2 (AP2)/ethylene-responsive element–binding proteins (EREBP) are one family of TFs, which in Arabidopsis contains 145 members and shares the AP2 DNA-binding domain of 70 amino acids originally identified in the floral homeotic protein APETALA2. The AP2/EREBP family is further divided into five subfamilies, AP2, RAV, DREB, ERF and others, based on the number of the AP2 domains presented in the genes. The DREB subfamily contains 56 members classified into six groups (A-1 to A-6) [1]. All of the members contain a single AP2 domain and bind to a consensus sequence (CCGAC) of the C-repeat or dehydration response element (DRE) in the promoters of genes that are turned on in response to low temperatures and/or water deficit. The DREB TFs were among the first TFs discovered to correlate with drought stress [2]. Over-expression of DREB TFs in transgenic plants remarkably increases the ability of plants to survive under strong drought and cold conditions [2]. The DREB TFs are also responsive to salinity stress [2]. All these functions are achieved by activating expression of their target genes, which include other TFs, phospholipase C, RNA-binding proteins, sugar transport protein, desaturase, carbohydrate metabolism related proteins, osmoprotectant biosynthesis proteins, proteinase inhibitors, late embryogenesis abundant (LEA) proteins (also known as dehydrins (DHNs)) and cold-responsive (COR) proteins [3]–[4]. DREB TFs can be induced by drought or by other DREB members [2].

MicroRNAs (miRNAs) are another class of important gene regulators, which can cleave a specific mRNA based on reverse sequence complementarities between the miRNA and target mRNA. MiRNAs regulate a wide range of biological processes including abiotic stresses. They are single-stranded non-coding (nc) small RNAs (sRNAs) between 20–24 nucleotides (nt) in length, which are processed from hairpin precursors (pre-miRNAs) by the Dicer-Like 1 enzyme (DCL1). Pre-miRNAs are derived from primary transcripts (pri-miRNAs) transcribed from genomic DNA, which is most likely to be regulated by TFs. On the other hand, TFs are possible miRNA targets. This reciprocal regulation has been shown in animals, which forms feedback loops [5]. In animals, miRNAs and TFs can regulate the same targets to form feed-forward loops [5]. However, in plants availability of data is limited.

Small interfering RNAs (siRNAs) are the second class of small regulatory RNAs. They are similar in size to miRNAs, but are processed from perfect long double-stranded RNAs derived from viruses, transgenes or endogenous transcripts such as transposons. Endogenous siRNAs are further classified into trans-acting siRNAs (tasiRNAs), natural antisense transcript-derived siRNAs (natsiRNAs), repeat-associated siRNAs (rasiRNAs), long siRNAs (lsiRNA) and heterochromatin siRNAs. The majority of siRNAs are 24 nt and 21 nt in length. 24-nt siRNAs mainly guide silencing at the transcriptional level, while 21-nt sRNAs mainly guide silencing at the post-transcriptional level. siRNAs can direct DNA methylation, which is particularly important in the regulation of transposable elements (TEs) that account for a large portion of the genome size and are a major driver of evolution. natsiRNAs have been demonstrated to regulate salt tolerance [6], disease resistance [7] and a ubiquitous TF, nuclear factor Y (NF-Y) [8] that contributes to drought tolerance in Arabidopsis and maize (*Zea mays* L.) [9].

Piwi-interacting RNA (piRNA) is a distinct class of ncsRNAs, which is found to be expressed only in animal cells [10]. Unlike miRNA and siRNA, piRNAs size between 23–29 nt, lack of sequence conservation and are processed through Dicer-independent mechanisms [10]. piRNAs function mainly in silencing TEs, but its pathway is at the forefront of defence against transposons in cells, which is achieved through association with PIWI proteins. piRNA and PIWI proteins form an active piRNA-induced silencing complex (piRISC) used to recognize and silence complementary RNA targets [10].

Other sRNA classes recently discovered include tiny ncRNAs (tncRNAs), 21U-RNA, scan RNA (scnRNA), promoter/termini-associated sRNAs (PASRs/TASRs), transcription initiation RNAs (tiRNAs), transcription start site-associated RNAs (TSSa RNAs), splice site RNAs (spliRNAs) and sRNAs derived from rRNA, snoRNA and tRNA [11]–[12]. Most of these sRNAs are generated through the miRNA/siRNA pathway [12]. tRNA-derived sRNAs (tsRNAs) are similar in function and biosynthesis to miRNAs/siRNAs, but essentially restricted to the cytoplasm and associated with Argonaute proteins. So far, 100 miRNAs have been identified in barley (*Hordeum vulgare* L.) [13], but the effect of TF on the expression of miRNAs and sRNAs in barley has not been investigated.

Barley is one of the most important cereal crops worldwide, ranking fourth in terms of production. Recently, transformation with a DREB TF (*TaDREB3*) from wheat (*Triticum aestivum* L.) driven by a duplicated 35S promoter has been shown to confer strong tolerance to drought and cold in transgenic barley (*H. vulgare* cv. Golden Promise) [14]. Further study revealed that the over-expression of *TaDREB3* in transgenic barley leads to elevated expression of other CBF/DREB genes and LEA/COR/DHN genes known to be responsible for the protection of cells from damage and desiccation under stress conditions [14]. In this study, we performed deep sequencing of sRNAs from a transgenic barley line expressing *TaDREB3* and from non-transgenic control barley. Comparative analysis showed that many group-specific and differentially expressed sRNAs existed between the two groups, including miRNAs and other classes of sRNAs. This indicates that the over-expressed TF affects the expression of various classes of sRNAs. Our data provides a valuable resource for identifying sRNAs related to the expression of DREB TFs in barley, whose outcome would help design a new effective strategy to cope with drought stress that severely limits the crop productivity.

## Results

### Drought Tolerance of Transgenic Barley Over-expressing *TaDREB3*

**Figure 1:**
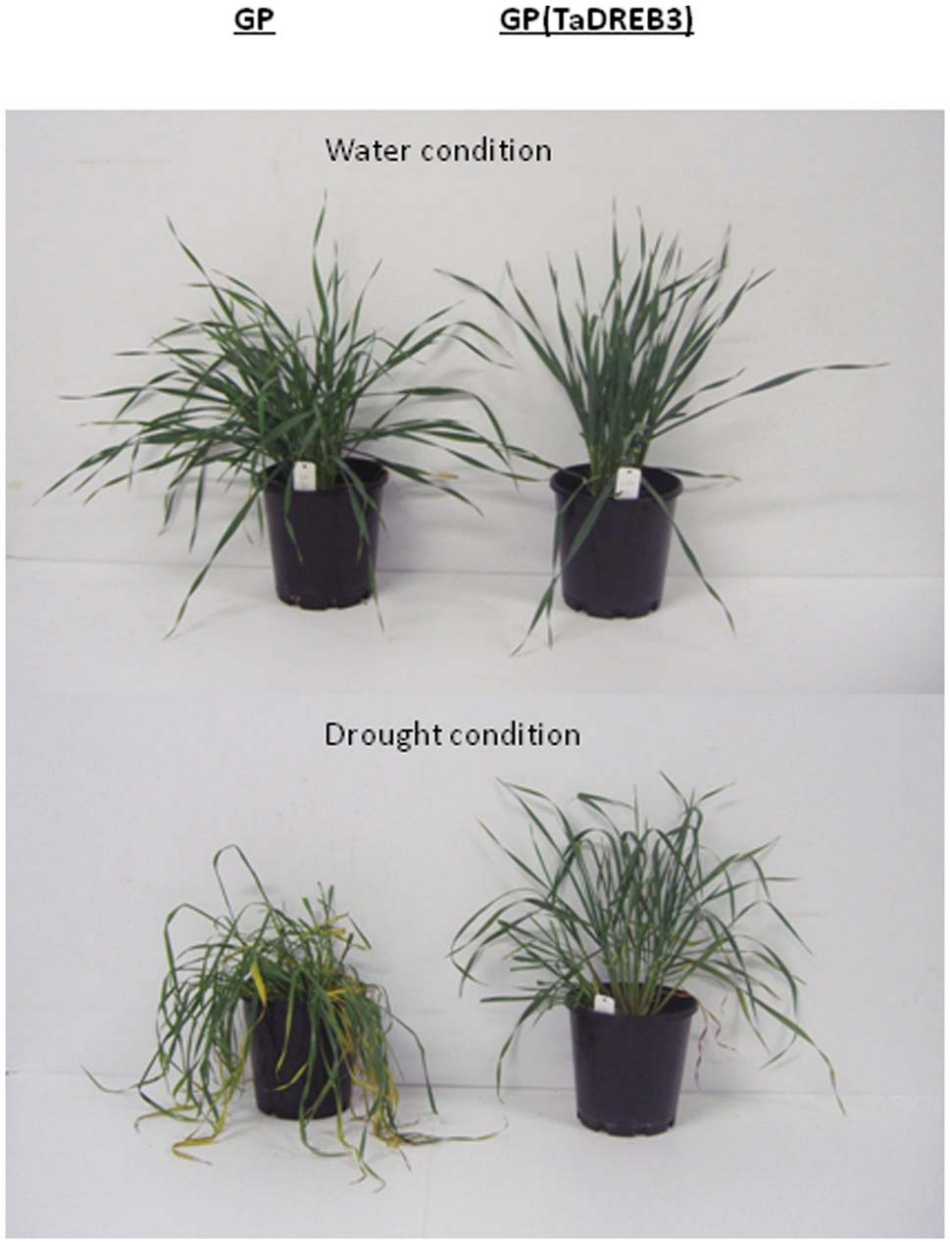
Phenotypes of non-transgenic GP and transgenic GP over-expressing TaDREB3 treated with drought and well water. The drought treatment was imposed by withholding water for seven days after the plants were grown for four weeks. TaDREB3 confers drought tolerance to transgenic barley.

T3 generation of transgenic barley lines with *TaDREB3* was evaluated for drought tolerance. Non-transgenic barley plants were used as a control. Both transgenic and non-transgenic plants were deprived of water for a week. After this treatment, distinct differences were observed between the transgenic and non-transgenic plants. The transgenic plants looked vigorous, while the non-transgenic plants were severely wilted (Figure 1). Analysis of water content in the plants showed that the transgenic plants contained more water than the non-transgenic plants. These results are consistent with previous studies [14]–[15]. The most drought-tolerant transgenic barley plants with *TaDREB3* and the most drought-intolerant non-transgenic barley plants were selected for further analysis of expression profiles of miRNAs and other classes of sRNAs.

### Sequencing of sRNAs from Non-transgenic and Transgenic Barley

Isolation and precise quantification of sRNAs are two keys for the comparison of different sRNA expression profiles. We used the Trizol reagent (Invitrogen) to extract total RNA from leaf tissues and the Purelink miRNA Isolation Kit (Invitrogen, Carlsbad, CA, USA) to isolate sRNAs to ensure good quality sRNAs for downstream gel purification. In addition, we adjusted the RNA preparations to the same concentration for ligation with the 5′ and 3′ adapters and for RT-PCR. Furthermore, we ran the RT-PCR products in the same flow cell for sequencing on the Illumina Genome Analyser so that we can assume the same sequencing error rate in both groups. These measures minimised artificial differences. In the end, we obtained 6,818,851 reads of 36 bases from transgenic barley and 7,107,714 reads of 36 bases from non-transgenic barley after filtering low quality reads. Both numbers are similar to each other. Reliable endogenous non-transgenic, constitutively expressed U6 spliceosome RNA [16] was perfectly parallel in read number between the two groups, indicating that a significant bias did not exist between groups. This thus allowed for the comparison of sRNA profiles between the two groups, and analysis of the effect of *TaDREB3* on the expression of sRNAs.

### Size Distribution of sRNAs in Non-transgenic and Transgenic Barley

**Figure 2:**
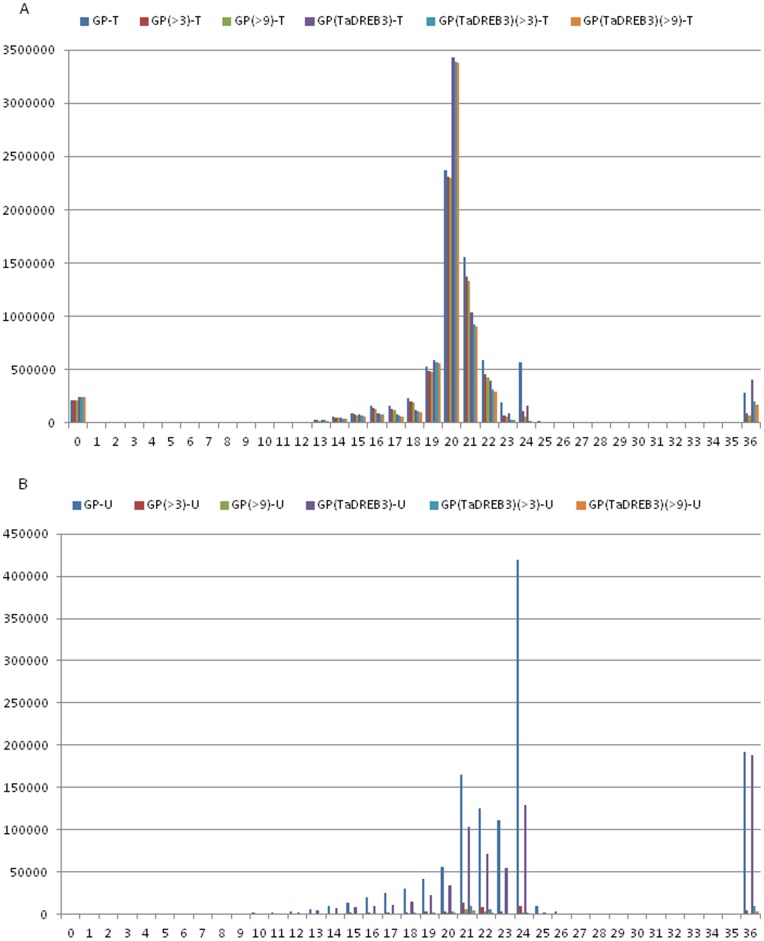
Size distribution of sRNA sequencing reads from non-transgenic barley and transgenic barley without a read count threshold, with a threshold > = 4 and with a threshold > = 10. A) Read count as a function of the read length. B) Number of unique reads as a function of the read length.

The size distribution of the adapter trimmed reads from transgenic and non-transgenic barley is specified in Tables 1 and 2, and displayed in Figure 2A. The read size with the highest expression values (read count) was 20 nt followed by 21 nt in both groups, which is consistent with the Dicer cleavage products. However, the read count of 21 nt in transgenic barley was much lower than in non-transgenic barley. By contrast, the read count of 20 nt was much higher in transgenic barley compared to non-transgenic barley despite the fact that the total read count in non-transgenic barley was slightly higher than in transgenic barley. The third most expressed length was 22 nt in non-transgenic barley and 19 nt in transgenic barley. 22-nt reads in transgenic barley were ranked fourth, while 19-nt reads in non-transgenic barley ranked fifth, in read count. The fourth abundant size of reads in non-transgenic barley was 24 nt, which was at the fifth position in transgenic barley. The 19, 22 and 24 nt were very similar in read count to each other in non-transgenic barley. However, in transgenic barley these sizes were different in read count: 19-nt reads were more abundant than 22-nt reads, whereas 22-nt reads were more abundant than 24-nt reads.

**Table 1:** Size distribution of reads from non-transgenic barley.

	threshold = 1		threshold> = 4		threshold> = 10	
read length	unique reads	read count	unique reads	read count	unique reads	read count
0	1	210867	1	210867	1	210867
1	4	874	4	874	3	868
2	13	1223	5	1208	2	1195
3	19	26	0	0	0	0
4	31	77	3	44	3	44
5	75	106	3	25	1	14
6	176	407	7	196	2	168
7	438	985	29	506	8	384
8	1057	3101	55	1927	19	1743
9	1267	3474	86	2105	33	1833
10	1613	3880	109	2116	45	1765
11	2190	7228	182	4813	75	4248
12	3420	12710	348	9030	118	7765
13	5979	31843	681	25416	282	23157
14	9523	62696	1209	52573	502	48597
15	14217	92360	1678	77158	727	71842
16	20428	162361	2304	140820	963	133294
17	24845	158839	2350	132272	1037	124822
18	30838	234259	2628	201421	1129	192880
19	42079	531625	3170	486882	1428	477046
20	56585	2370690	4095	2310477	1746	2297334
21	165048	1554458	13429	1377789	5823	1335020
22	125795	593035	9028	457753	3630	427570
23	111309	192190	2952	71856	882	60608
24	419485	569003	10005	105823	1890	63240
25	10140	18330	315	7553	101	6386
26	2938	4662	97	1532	33	1182
27	0	0	0	0	0	0
28	0	0	0	0	0	0
29	0	0	0	0	0	0
30	0	0	0	0	0	0
31	0	0	0	0	0	0
32	0	0	0	0	0	0
33	0	0	0	0	0	0
34	0	0	0	0	0	0
35	0	0	0	0	0	0
36	191990	286405	4249	88532	1439	72969
total reads	1241503	7107714	59022	5771568	21922	5566841

**Table 2:** Size distribution of reads from transgenic barley.

	threshold = 1		threshold> = 4		threshold> = 10	
read length	unique reads	read count	unique reads	read count	unique reads	read count
0	1	244887	1	244887	1	244887
1	4	740	4	740	3	736
2	9	1222	2	1210	1	1203
3	8	11	0	0	0	0
4	12	17	1	5	0	0
5	29	32	0	0	0	0
6	12	13	0	0	0	0
7	19	34	1	16	1	16
8	33	47	2	12	0	0
9	75	117	5	34	1	11
10	265	603	22	329	9	263
11	888	3860	77	2912	34	2686
12	2423	10805	248	8141	81	7201
13	5025	31084	649	25642	260	23435
14	7196	49421	1126	41845	453	38029
15	8978	76421	1354	66927	556	62402
16	10078	91358	1471	80632	595	75693
17	10810	76782	1531	65399	629	60236
18	14449	123565	1932	108444	839	102136
19	23146	590427	2712	566325	1245	557986
20	34549	3427405	3449	3390972	1584	3380370
21	104213	1041397	9622	930122	4201	899662
22	71319	391368	5674	314424	2343	295743
23	54931	88444	1394	29446	438	24166
24	129638	162602	2107	21354	378	12363
25	1942	4039	77	1951	29	1669
26	542	823	18	242	7	177
27	0	0	0	0	0	0
28	0	0	0	0	0	0
29	0	0	0	0	0	0
30	0	0	0	0	0	0
31	0	0	0	0	0	0
32	0	0	0	0	0	0
33	0	0	0	0	0	0
34	0	0	0	0	0	0
35	0	0	0	0	0	0
36	188803	401327	9972	203298	3649	167962
total reads	669397	6818851	43451	6105309	17337	5959032

After grouping the total reads, 445,672 and 989,288 unique reads remained in transgenic and non-transgenic barley, respectively (Tables 1 and 2). The unique read number was over twice in non-transgenic barley than in transgenic barley. Figure 2B shows size distribution of the unique reads. 24 nt was the most abundant read length followed by 21 nt in both groups. This, combined with the above size distribution of the read counts, indicated that 21-nt, especially 20-nt, reads comprised many highly expressed reads (high abundance), while 24-nt reads consisted primarily of unique or low abundant reads, which is in line with previous studies. This in turn demonstrated that the sequencing result from each group was reliable and each read could thus represent its relative abundance *in vivo*.

Comparative analysis showed that in non-transgenic barley the number of 24-nt unique reads was more than twice of 21-nt unique reads. In contrast, in transgenic barley the numbers of 24-nt and 21-nt unique reads were similar to each other. Moreover, 24-nt unique reads were more than twice in non-transgenic barley than in transgenic barley. To check the abundance of unique reads as a function of its length, we applied a threshold to the read count of 4 (limit is included). Although the numbers of reads with sizes less than 20 nt or greater than 24 nt were not obviously reduced in the two groups, the numbers of 20–24-nt reads were significantly lower, especially in non-transgenic barley (Figure 2B), thereby bringing the unique read numbers of the two groups back into approximation. This result demonstrated that more low abundant reads existed in non-transgenic barley than in transgenic barley (this is further confirmed when setting the threshold to 10). It is to note that we cannot exclude the possibility that a portion of the low abundant reads may be due to sequencing errors. However, the sequencing error should have the same magnitude in both samples as they have been run in the same flow cell. This means that we can assume that the existence of a lower number of low copy reads (specially 24 nt long reads) in transgenic barely is a direct consequence of the TF.

### sRNAs Derived from Chloroplast and Nuclear in Transgenic and Non-transgenic Barley

Previous studies showed that a number of Illumina sequencing reads map to chloroplast genomes [13], [17]–[19]. These reads were classified into chloroplast-derived sRNAs (csRNAs), which have been proposed to play roles in abiotic stress tolerance [20]. To categorize chloroplast and nuclear sRNAs, we mapped all the trimmed reads to the complete barley chloroplast genome sequence [21] by means of the Bowtie aligner. Our analysis revealed that 3322007 reads represented by 23477 unique reads in transgenic barley and 2651785 reads represented by 30387 unique reads in non-transgenic barley mapped to the barley chloroplast genome (Table 3). These reads were labelled as chloroplast derived reads. Correspondingly, the unmapped reads were labelled as nuclear derived reads or more precisely, non-chloroplast reads as the nuclear genome also contains the chloroplast-derived sequences [22]. It is expected that some chloroplast sRNAs (csRNAs) may be of nuclear origin because the sRNA processing machinery most likely resides in the nucleus.

**Table 3:** Statistics of reads from Golden Promise barley (GP) and Golden Promise transgenic barley over-expressing *TaDREB3* [GP(TaDREB3)].

non-chloroplast reads						chloroplast reads (0 mismatches)				
> = 1	GP		GP(TaDREB3)	fraction		> = 1	GP	GP(TaDREB3)	fraction	
unique reads	958901		422195	2.271228		unique reads	30387	23477	1.294331	
total read count	3575645		2585109	1.38317		total read count	2651785	3322007	0.798248	
sample specific reads	865500		328794	2.632347		sample specific reads	20673	2545	8.122986	
common reads	93401			log2 sum	log2 fraction	common reads	5922		log2 sum	log2 fraction
|log2| > = 1	18346	15477		33823	36.21267	|log2| > = 1	1252	1348	2600	43.90409
> = 4	GP	GP(TaDREB3)		fraction		> = 4	GP	GP(TaDREB3)	fraction	
unique reads	44148	24695		1.78773		unique reads	3929	3828	1.026385	
total read count	2535876	2132417		1.189203		total read count	2617582	3296389	0.794076	
sample specific reads	10367	4252		2.438147		sample specific reads	555	181	3.066298	
common reads	42333			log2 sum	log2 fraction	common reads	3518		log2 sum	log2 fraction
|log2| > = 1	13899	9897		23796	56.21147	|log2| > = 1	1252	851	2103	59.77828
> = 10	GP	GP(TaDREB3)		fraction		> = 10	GP	GP(TaDREB3)	fraction	
unique reads	16260	10123		1.606243		unique reads	1442	1574	0.916137	
total read count	2382398	2050931		1.161618		total read count	2603761	3283687	0.792938	
sample specific reads	859	286		3.003497		sample specific reads	42	29	1.448276	
common reads	18293			log2 sum	log2 fraction	common reads	1736		log2 sum	log2 fraction
|log2| > = 1	6222	3431		9653	52.76882	|log2| > = 1	670	434	1104	63.59447

The ratio of the chloroplast reads and non-chloroplast reads is 1.29∶1 in transgenic barley and 0.72∶1 in non-transgenic barley (Table 3). The chloroplast reads were more in transgenic barley and less in non-transgenic barley than the non-chloroplast reads. However, the chloroplast unique read number in both groups was much smaller than the non-chloroplast unique read number (Table 3), indicating that the chloroplast reads are highly redundant.

The non-chloroplast unique read and total read numbers in non-transgenic barley were 2.27 and 1.38 times more, respectively, than those in transgenic barley (Table 3). In order to compare the two groups, we multiplied the read counts of transgenic barley by a factor of 1.38, which generated identical total read counts in both groups. Cross comparison showed that in transgenic barley 328,794 non-chloroplast unique reads were specific, accounting for 77.9% of the total unique reads in the plant, while in non-transgenic barley 865,500 non-chloroplast unique reads were specific, accounting for 90.3% of the total unique reads in the plant (Table 3). However, when a threshold of 10 was set, the number of group specific reads was reduced by a factor of over 1000. In contrast, the number of shared reads (detected in both groups) was only reduced by a factor of 5. 36.2% of the shared reads showed a log2-ratio of |log2| > = 1 that corresponds to a differential expression between the two groups of at least a factor of 2, i.e. twice as high in one group compared to the other (Table 3). Interestingly, the fraction of differentially expressed reads increased when setting a threshold (Table 3). These data indicate that the over-expressed TF affects the expression of both nuclear and chloroplast sRNAs in the plant.

### Expression Profile of Known miRNAs in Transgenic and Non-transgenic Barley

To detect known miRNA sequences with miRanalyzer [23], all the non-chloroplast reads shorter than 17 bases were removed from our datasets. The remaining reads were first mapped (without mismatch) to known barley miRNAs in the miRBase (miRBase version 17, April 2011). Nineteen out of the 22 barley miRNAs annotated in the miRBase were detected in non-transgenic barley and 17 detected in transgenic barley (Table 4). The two extra miRNAs in non-transgenic barley were huv-miR444a and huv-miR1126 (Table 4).

**Table 4:** Mapped barley miRNAs in miRBase.

miRNA	miRNA sequence	GPread count	GP(TaDREB3)read count	GP(TaDREB3)normalised	log2
hvu-miR5048	UAUUUGCAGGUUUUAGGUCUAA	4547	2106	2912.955844	−0.642431171
hvu-miR159a/bhvu-miR159a*	UUUGGAUUGAAGGGAGCUCUGGAGCTCCTATCATTCCAATGA	22465	42602	5892.303845	1.391473899
hvu-miR444b	UGCAGUUGCUGUCUCAAGCUU	64	30	41.4950975	−0.625131008
hvu-miR1120	ACAUUCUUAUAUUAUGGGACGGAG	387	94	130.0179722	−1.573622508
hvu-miR444a	UUGCUGCCUCAAGCUUGCUGC	1	0	0	#NUM!
hvu-miR156	UGACAGAAGAGAGUGAGCACA	140769	213440	295223.787	1.068479308
hvu-miR1436	ACAUUAUGGGACGGAGGGAGU	8	2	2.766339833	−1.532021604
hvu-miR5050	UUGAGGUCGUUCAACCAGCAA	57	27	37.34558775	−0.610024116
hvu-miR5051hvu-miR5051*	UUUGGCACCUUGAAACUGGGACCAGTTTCAAGGTTTCAAAGC	41	50	6.915849583	0.789906491
hvu-miR171	UGAUUGAGCCGUGCCAAUAUC	158	88	121.7189527	−0.376370733
hvu-miR168-3phvu-miR168-3p*	GAUCCCGCCUUGCACCAAGUGAAUCCCGCCTTGCACCAAGTGAAT	404324	293248	405.2687856	0.004523768
hvu-miR399	UGCCAAAGGAGAUUUGCCCCG	2	1	1.383169917	−0.532021604
hvu-miR5049	UCCUAAAUACUUGUUGUUGGG	549	121	167.3635599	−1.713820705
hvu-miR397	CCGUUGAGUGCAGCGUUGAUG	8	7	9.682189416	0.275333318
hvu-miR166/b/c	UCGGACCAGGCUUCAUUCCCC	19883	6967	9636.544809	−1.044947586
hvu-miR1126	UCAACUAUGGACUACAUACGGAA	13	0	0	#NUM!
hvu-miR5052	ACCGGCUGGACGGUAGGCAUA	11	3	4.14950975	−1.406490722
hvu-miR168-5p	UCGCUUGGUGCAGAUCGGGAC	420777	179792	248682.8857	−0.758748623
hvu-miR5053	CGCAGCUGUAGUCGCCGGCGU	24	5	6.915849583	−1.795056009

Note: GP-Golden Promise barley;

GP(TaDREB3)-Golden Promise transgenic barley over-expressing *TaDREB3;*

miRNA*, star sequence and read count are highlighted in blue.

hvu-miR156 was the most abundant miRNA in transgenic barley, accounting for 52.4% of the total reads, and the second most abundant in non-transgenic barley, while hvu-miR168-5p was the most abundant in non-transgenic barley, accounting for 71.3% of the total reads, and the second most abundant miRNA in transgenic barley (Table 4). Both miRNAs together accounted for 95.2% of the total miRNA reads in non-transgenic barley and 96.6% in transgenic barley (Table 4). When threshold > = 10 was set, 14 barley miRNAs in non-transgenic barley and 13 in transgenic barley retained. The extra miRNA in non-transgenic barley was huv-miR1126.

To detect previously un-annotated mature* sequences, which are determined by calculating the theoretical mature* sequence in the secondary structure assuming a perfect 3′ overhang of 2 nt, all the reads were aligned to the pre-miRNA sequences. Three novel mature* sequences were detected (Table 4, highlighted in blue). It is worth mentioning that the annotated hvu-miR168-3p sequence in the miRBase might be wrong, because firstly the annotated sequence does not form an overhang with the mature (hvu-MIR168-5p) sequence in the secondary structure, and secondly the theoretical mature* sequence coincides with the most expressed read that map to the 3p arm in both groups. Apart from the detected mature* sequences, isomiRs of many barley miRNA were also detected in the two groups (Table 5). These isomiRs have their 5′ or 3′ ends longer or shorter than, or one nt different from, their reference miRNA sequences (data not shown).

**Table 5:** Detected isomiRs of barley miRNAs in Golden Promise barley (GP) and Golden Promise transgenic barley over-expressing *TaDREB3* [GP(TaDREB3)].

GP					GP(TaDREB3)			
miRNA	uniquereads	readcount	norm_expressed_all	norm_expressed_mapped	miRNA	uniquereads	readcount	norm_expressed_all
hvu-MIR5048	122	2222	0.031261809	16.62924712	hvu-MIR5048	62	1006	0.014753219
hvu-MIR5052	28	511	0.007189372	3.824277803	hvu-MIR159a	20	144	0.002111793
hvu-MIR159a	43	211	0.002968606	1.579104924	hvu-MIR5052	5	95	0.001393197
hvu-MIR5050	10	132	0.001857137	0.987876066	hvu-MIR444a	8	44	6.45E-04
hvu-MIR444a	8	109	0.001533545	0.815746146	hvu-MIR168	5	35	5.13E-04
hvu-MIR168	10	62	8.72E-04	0.464002395	hvu-MIR444b	10	30	4.40E-04
hvu-MIR444b	19	47	6.61E-04	0.351743751	hvu-MIR5049	11	25	3.67E-04
hvu-MIR171	7	39	5.49E-04	0.291872474	hvu-MIR5050	5	24	3.52E-04
hvu-MIR1126	25	35	4.92E-04	0.261936836	hvu-MIR171	8	20	2.93E-04
hvu-MIR1120	6	34	4.78E-04	0.254452926	hvu-MIR1120	3	14	2.05E-04
hvu-MIR5049	14	26	3.66E-04	0.194581649	hvu-MIR1126	10	12	1.76E-04
hvu-MIR1436	6	15	2.11E-04	0.112258644	hvu-MIR1436	2	5	7.33E-05
hvu-MIR397	5	14	1.97E-04	0.104774734	hvu-MIR5053	3	3	4.40E-05
hvu-MIR5053	4	10	1.41E-04	0.074839096	hvu-MIR397	2	2	2.93E-05
hvu-MIR156	4	4	5.63E-05	0.029935638	hvu-MIR156	2	2	2.93E-05
hvu-MIR5051	2	2	2.81E-05	0.014967819				

Next, we removed the reads mapping to barley miRNAs and aligned the remaining reads (without mismatch) to known miRNAs from other species in the miRBase. A total of 126 miRNAs in transgenic barley and 150 in non-transgenic barley were mapped, of which 25 and 49 were specific to transgenic and non-transgenic barley, respectively, while 101 were common between the two groups (Table S1). Of these miRNAs, 23 are the origin of animals, of which 14 were specific to non-transgenic barley, 7 were specific to transgenic barley and 7 were common between the two groups. miRNAs conserved between plants and animals had been the case before [24] and could be used as a phylogenetic marker to discover the evolutionary history and pattern between plants and animals. When threshold > = 10 was applied, 65 miRNAs in non-transgenic barley and 64 in transgenic barley remained (Table S1). vvi-miR172d (or ata-miR172) is only present in non-transgenic barley. With this threshold, only one putative animal miRNA (dme-miR1-3p) remained in the two groups, indicating that most of the animal miRNAs are low abundant. Interestingly, this putative animal miRNA was up-regulated in transgenic barley and had its targets found in barley (Table 6). Whether it is indeed associated with the TF is not clear. All the detected non-barley miRNAs are considered as homologous miRNAs.

**Table 6:** Up-regulated miRNAs in Golden Promise transgenic barley over-expressing *TaDREB3*.

miRNA	target accession	known annotation	new annotation
hvu-miR156/zma-miR156k/sbi-miR156e/ptc-miR156k/vvi-miR156a/h/ath-miR156g/smo-miR156a/aly-miR156g	TC207028 TC237973 TC195116 TC219375 TC218364 TC195122 TC195124 TC200044 TC225150 TC210261 TC223315 TC202631 TC222091 CA032492 BF258419 EX598089 TC209643 TC203114 TC232888 BF630636 TC219515 BQ766747 TC219782 BE421183 TC233676 TC203114 BQ767847 DN156768 TC198370 TC215757 TC198750 BQ469258	Squamosa promoter-binding- likeprotein	unknown protein,resistance protein,protein kinase,F-box domain containing protein,teosinte glume architecture 1,promoter binding protein
hvu-miR159b/a	TC216219 TC195133 CV062223 TC208671	MYB transcriptionfactor	60S ribosomal protein L30triosephosphate isomerase
osa-miR395b/d/e/g/h/i/j/k/l/s/m/n/p/q/r/ysbi-miR395b/a/d/e/c/g/h/i/jzma-miR395b/a/d/e/f/g/h/i/j/n/ptae-miR395a	TC219499 BG344292 TC200840 BE602779 BQ756651 TC199938	ATP sulphurylases	unknown protein,magnesium-protoporphyrin IX monomethyl ester [oxidative] cyclase
dme-miR1-3p/dps-miR1/ame-miR1/aga-miR1/bmo-miR1a/tca-miR1-3p/dan-miR1/der-miR1/dgr-miR1/dmo-miR1/dpe-miR1/dse-miR1/dsi-miR1/dvi-miR1/dwi-miR1/dya-miR1/dpu-miR1/isc-miR1/aae-miR1/cqu-miR1/api-miR1/nvi-miR1/ngi-miR1	TC205060 TC216030 TC222124 TC205544 TC225866 CB866465 CX631822 BI954943	Delta encoding amembrane-boundligand in animals	unknown protein,Gamma-thionin,S-like RNase,polyprotein,fructose-bisphosphate aldolase

**Figure 3:**
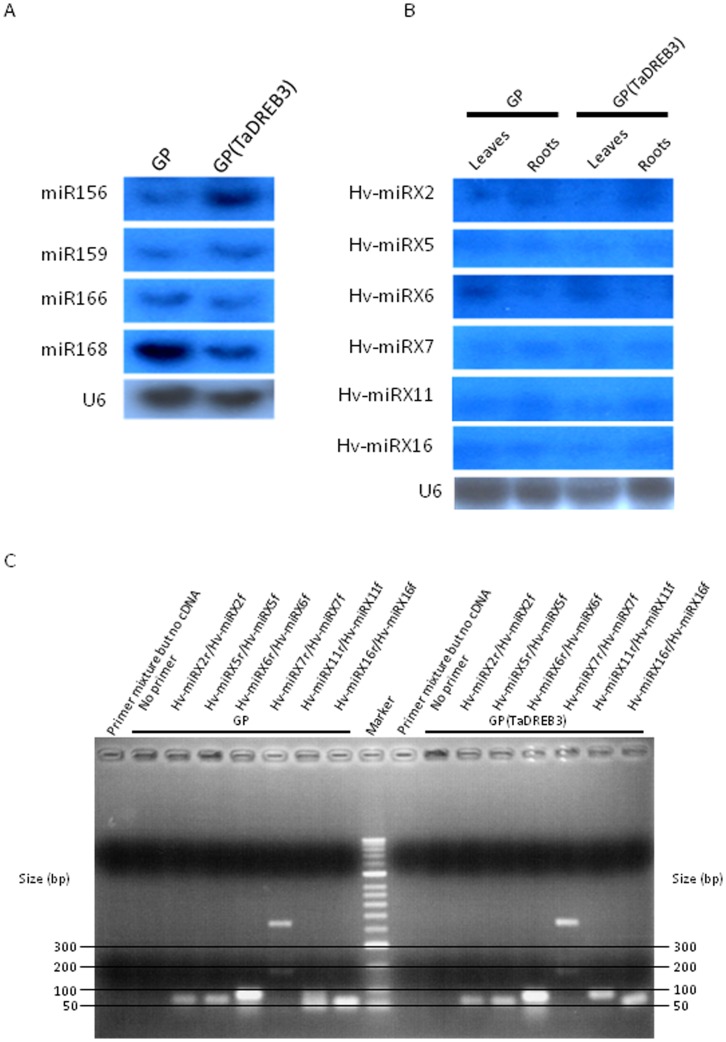
Analysis of miRNAs by Northern blot hybridization and RT-PCR. Total RNA was hybridized with probes for known miRNAs (A) and putatively novel miRNAs (B) and RT-PCR with primers specific for putatively novel miRNAs (C) that are shown in Table S2. U6 in (A) and (B) was used as a loading control. Reactions without cDNA templates or primers were used as controls. Sizes of 100-bp DNA ladder (Bioline) are indicated.

After the normalization of read count, 4 miRNA families were shown to be significantly up-regulated (log2> = 1) (Table 6), while 24 miRNA families were significantly down-regulated (log2< = −1), in transgenic barley (Table 7). Some of these differentially expressed miRNAs were further confirmed by Northern hybridization (Figure 3A). Many differentially expressed miRNAs target transcription factors related to plant development, morphology and flowering time (Tables 6 and 7). Other differentially expressed miRNAs target the genes involved in plant metabolic metabolisms (succinyl-CoA ligase beta-chain, potassium transporter, NADH dehydrogenase), stress response (protein kinase, resistance protein) and post-translational modifications (ribosomal protein, F-box protein) (Tables 6 and 7). Remarkably, differentially expressed miR172 targets the AP2 subfamily, a close relative of the DREB subfamily. Both AP2 and DREB subfamilies belong to the AP2 TF family.

**Table 7:** Down-regulated miRNAs in Golden Promise transgenic barley over-expressing *TaDR EB3.*

miRNA	Target Accession	known annotation	New annotation
osa-miR164d/sbi-miR164b/zma-miR164f/bdi-miR164c/far-miR164a/b	TC196726 TC205144 TC208432 TC211596 TC213127DN183772 DN156119 TC228953 TC226313 TC226106	NACtranscriptionfactor (TF)	unknown protein, protein kinase, sperm protamine-P1,phytosulfokine-alpha 1 precursor
miR166	CV063912 TC231714 TC204606	HD-ZIPIII TF	unknown protein
tae-miR167b	DN178141 DN156631 TC228910 TC222640	Auxinresponse TF	unknown protein, protein kinase Nek5
sof-miR168b	TC216443 TC205587 NP315934	Argonaute1	unknown protein, fowl adenovirus D, flame chlorosis virus-like agent
miR169	TC205164 TC211039 TC199079	CCAAT-binding TF	unknown protein, SRPK4,
vvi-miR172d/ata-miR172	TC235219 TC199223 TC196893 TC223285 AL499992AL501298 BQ464727 TC229495 TC200442 BM442236TC206014 TC206508 TC208243 CD053703	APETALA2 TF	unknown protein, Sporulation related precursor, succinyl-CoA ligase beta subunit, squalene monooxygenase flagellar hook-associated protein FlgK precursor,
zma-miR319b*/d*	TC197194 BF260639 TC223199 TC223334 TC229714BG343280 BE454497	TCP TF	unknown protein, 40S ribosomal protein S15a-1
zma-miR396f*/osa-miR396f-3p	BJ458177 CK568233 TC221734 TC220586 TC214896TC227263	Growth-regulating TF	unknown protein, ribosomal protein S13e, glycin-rich protein,Kelch repeat-containing F-box-like protein
osa-miR827a/b/zma-miR827/bdi-miR827/ssp-miR827	BJ470052 GH217971 TC221516 BJ468837 TC197379EX575934	SPX protein	unknown protein, T2P11.4 protein, ATP-dependent Clp protease ATP-binding subunit, aberrant pollen transmission 1
hvu-miR1120	CK566710 TC196415 TC227275 TC201904 TC211215TC212619 BE231018 TC203981	Not available	unknown protein, TAK14
osa-miR1426	TC211645 TC227152 BQ464676 BF065989 TC208577DN183702 TC236776 TC237637	Not available	unknown protein, ADR183Cp, Receptor-like kinase
hvu-miR5049	TC211425 TC203435 TC210715 BI779287 DN158159TC208187 BM101048 TC198208	Not available	unknown protein, tubby protein-like, non-specific lipid-transfer protein, resistance-related receptor-like kinase,Lipase class 3-like, farnesyl pyrophosphate synthase
hvu-miR5052		Not available	no result
hvu-miR5053	TC222348 TC204313 TC197442 TC229752 BE214070BF264468 BI951909 TC228474 BE213779 BF628941BG299890 TC232455 BI956149 BE411948	Not available	Unknown protein, Myb-related protein,chlorophyll a/b-binding protein CP29 precursor, AGAP011446-PA
bdi-miR5054		Not available	no result
bdi-miR5062a/b	TC199433 CA014897	Not available	unknown protein
osa-miR5072		Not available	no result
osa-miR5083	TC202028	Not available	unknown protein
tae-miR5084	TC196125 BY852481 BG365127	Not available	pyruvate dehydrogenase kinase isoform 1, potassium transporter
tae-miR5085	GH213771 AJ480573 TC207422 TC224281 TC200097TC199118 DN186570 CK124439 TC221808 TC215345	Not available	unknown protein, 23 kDa jasmonate-induced protein, NADH dehydrogenase subunit 2, OSE2-like protein, Nucellin-like aspartic protease
rgl-miR5139		Not available	no result
bdi-miR5169	BJ447165 TC223375 TC234516 GH210985 TC198932TC219591 BF256760 TC224793 DN157252 TC237517	Not available	unknown protein, probable 3-carboxymuconate cyclase protein,Surface antigen protein 2, cell death associated protein, DP protein
bdi-miR5200	TC210538 TC223555 TC202855 TC232508 TC208205	Not available	unknown protein, FT, FT-like protein

### Predicted Novel miRNAs and their Target Genes in Transgenic and Non-transgenic Barley

To identify novel miRNA candidates, the remaining unmapped reads were aligned to barley genomic or EST sequences. If some reads did not map the available barley genomic or EST sequences, these reads would then be aligned to genomic or EST sequences from the closely related species, wheat, Brachypodium, or rice. After examining protein-coding possibility, repetitive sequences and hairpin structures of mapped pre-miRNA sequences using the criteria as described [13], we identified 20 putative novel miRNAs in each group, of which 7 (Hv-miRX3, 7, 9, 10, 16, 18 and 20) were down-regulated, while 1 (Hv-miRX4) were up-regulated, in transgenic barley (Table 8). Two pairs of miRNAs (Hv-miRX7/Hv-miRX16, and Hv-miRX14/Hv-miRX15) were each derived from the same BAC clones. One miRNA (Hv-miRX19) localised between two repetitive sequences. Most predicted novel miRNAs were low abundant (Table 8). To experimentally confirm these putative miRNAs, northern hybridization was applied. All the selected miRNAs were detected in the leaf and root tissues (Figure 3B). It appeared that some miRNAs were expressed differently between the tissues or between the groups (Figure 3B). These results were further confirmed by RT-PCR (Figure 3C) with primers specific to their precursor miRNAs (Table S2), indicating that the predicted novel miRNAs are bona fide miRNAs.

**Table 8:** Predicted novel miRNAs in Golden Promise barley (GP) and Golden Promise transgenic barley over-expressing *TaDREB3* [GP(TaDREB3)].

miRNA	miRNA sequence	GPread count	GP(TaDREB3)read count	GP(TaDREB3)normalized	log2	hairpin MFE(kcal/mol)	location
Hv-miRX1	GAATGACGCCGGGTCCGAAAG	25	20	27.6633983	0.1460503	−204	dbj|AK252042.1|
Hv-miRX2	AGTGACGCGCATGAATGGATT	269	183	253.120095	−0.08778413	−143.1	dbj|AK248318.1|
Hv-miRX3	TGCTCACTGCTCTATCTGTCACC	5	1	1.38316992	−1.8539497	−169.5	dbj|AK372560.1|
Hv-miRX4	AGGTGTCATCCCGCCTGAACA	2	5	6.91584958	1.78990649	−152.6	dbj|AK370571.1|
Hv-miRX5	ACTGGTTGGATCATGCTTCTC	101	39	53.9436267	−0.90483087	−113.5	dbj|AK252755.1|
Hv-miRX6	ATTTACTTGTAGAAGAAGCTA	20676	14730	20374.0929	−0.02122128	−189.9	dbj|AK357352.1|
Hv-miRX7	TCAGATGAGAAGGCAGATCATA	135	41	56.7099666	−1.2512852	−210.4	dbj|AK364228.1|
Hv-miRX8	TAGGAAAGTAGAGTAGGCACA	6	5	6.91584958	0.20494399	−58.3	gb|HQ619243.1|
Hv-miRX9	ATTGACGACCTAGATACACGTGCA	77	15	20.7475487	−1.89191755	−60.1	gb|EF012202.1|
Hv-miRX10	TCTGTAACTTAATATAAGACG	33	8	11.0653593	−1.57641572	−137.4	BU991655.1
Hv-miRX11	TATTTGCAGGTTTTAGGTCTAA	3876	1806	2498.00487	−0.63379228	−43.1	dbj|AK248748.1|
Hv-miRX12	AATTATTTAGGTACAGAGGGA	42	20	27.6633983	−0.60241093	−146.2	dbj|AK367701.1|
Hv-miRX13	AATTAATATGGATCGGAGGGA	20	13	17.9812089	−0.15350998	−114.1	dbj|AK358446.1|
Hv-miRX14	CCGAACTGATGGAAAGGGCTA	46	17	23.5138886	−0.96812072	−75.8	gb|EU282577.1|
Hv-miRX15	TTTTGGTTGCGTTGGCTAGTGCAT	9	4	5.53267967	−0.70194661	−51.8	dbj|AK366688.1|
Hv-miRX16	TATAGTAATGATGGCTAATGGT	1149	263	363.773688	−1.6592657	−97.2	dbj|AK364228.1a|
Hv-miRX17	TTAGGATTAGGAATAGGTGTA	28	12	16.598039	−0.75441402	−65.2	gb|EF115541.1|
Hv-miRX28	AAATAGAATAATGATCAACGGA	22	3	4.14950975	−2.40649072	−37.1	gb|HQ619322.1|
Hv-miRX19	TTTGAGGGTTCTAGTCTTTGC	5	4	5.53267967	0.1460503	−117.9	gb|AF521177.1|
Hv-miRX20	ATCGTATGAACTTGAAGCAACGGT	50	11	15.2148691	−1.71644617	−119.5	gb|FJ477093.1|

To reveal the function of the predicted novel miRNAs, their targets were predicted using psRNATarget (http://bioinfo3.noble.org/psRNATarget). As is common for barley, most predicted targets were functionally unknown (Table S3). Of the function-known targets, many encoded protein kinases, suggesting that the corresponding miRNAs may be involved in signal transduction pathways especially in developmental processes. Only a few targets encode TFs. In contrast, the predicted targets of conserved miRNAs were enriched in TFs (data not shown). Most of the predicted novel miRNAs regulated the targets through cleavage (Table S3). However, 3 miRNAs regulated their targets through both cleavage and translational inhibition (Table S3). Moreover, although barley EST sequences are very limited, 18 out of the 20 miRNAs were found to target more than one gene (Table S3). In addition, one miRNA (Hv-miRX15) had two potential cleavage sites in one target (TC226135) (Table S3).

To validate the predicted miRNA targets, which could in turn confirm the predicted novel miRNAs, we constructed a degradome library based on the miRNA-directed cleavage between the 10th and 11th nt of complementarity relative to the guiding miRNA. If a target is cleaved by a miRNA, then its 3′ fragment having a 5′ monophosphate and a 3′ polyA tail can be ligated with an RNA adapter containing a 5′ MmeI restriction site. In contrast, full-length mRNAs cannot be ligated with this adapter as they are capped at the 5′ ends. After reverse transcription, second-strand synthesis and *Mme*I digestion, a 20–21-nt sequence of the mRNA plus the 5′ adapter remains, to which a 3′ dsDNA adapter was added and the library was amplified and then sequenced. In general, these 20−21nt signatures were sufficient to unambiguously identify the transcripts of origin and the corresponding miRNAs. As can be seen, the degradome library has advantages over the RNA ligase-mediated 5′ rapid amplification of cDNA ends (RLM 5′-RACE), which requires a priori miRNA target prediction and only tests a target at a time. We obtained a total of 5,270,019 sequences from the degradome library, of which 443,276 were unique. The sizes of sequences ranged from 13 to 26 nt, but the majority was between 20−21 nt. Alignment of the unique sequences with the predicted novel miRNAs revealed that all the novel miRNAs had perfectly reverse complementary sequences in the library (Table 9). While most cleavage sites took place between positions 10 and 11 with respect to the guiding miRNA, other cleavage sites occurred at positions 11–15 (Table 9). In addition, some miRNAs such as Hvu-miRX2 and Hvu-miRX5 can cleave their targets at different positions (Table 9). These data indicate that miRNA-mediated cleavage sites are diverse. Moreover, a few miRNAs can each be aligned to more than one sequence tag (Table 9), indicating that these miRNAs may have multiple targets. Because of the limited barley ESTs, we only found ESTs available for 6 sequence tags (Table 9). However, 4 of these 6 sequence tags are contained by more than one EST (Table 9). With more ESTs available, it is expected that more ESTs can be found to contain these sequence tags.

**Table 9:** Detected target sequences of novel miRNAs in barley degradome library.

miRNA	miRNA sequence	Target cleavage site	Target accession (0 mismatches)	Annotation
Hvu-miRX1	GAATGACGCCGGGTCCGAAAG	10/11: AGGCGTCATTCAAATTTCTG	Not available	Not available
Hvu-miRX2	AGTGACGCGCATGAATGGATT	10/11: GCGCGTCACTGCAACGGATAA10/11: GCGCGTCACTAATTAGATGAC12/13: ATGCGCGTCACTAATTAGATG13/14:CATGCGCGTCACTAATTAGAT	Not available for “GCGCGTCACTGCAACGGATAA”BY864760/BY865136/BY859056/BY865253/BY861299/BY839718/TC275169/BY864507/TC281249/AL508349/BI951089/TC279957/AJ432484/AU252321/TC281472/BI949016/TC281181/TC281158/TC257287/TC257303/BI953026/TC262239/TC278355/TC261623/TC268219/TC275184/TC274467/TC273938/TC268772	P450-like TBP,prolin rich protein,B hordein precursor,6-phosphogluconate dehydrogenase,decarboxylating,ribosome protein
Hvu-miRX3	TGCTCACTGCTCTATCTGTCACC	10/11: GCAGTGAGCAGCCGGGTTAA	Not available	Not available
Hvu-miRX4	AGGTGTCATCCCGCCTGAACA	10/11: GATGACACCTTTAGAGCGGG	dbj|AK356860.1|	unknown protein
Hvu-miRX5	ACTGGTTGGATCATGCTTCTC	10/11: TCCAACCAGTCAGATCCAACCA10/11: TCCAACCAGTCCGACGATCCAA11/12: ATCCAACCAGTCCGACGATCCA12/13: GATCCAACCAGTCCGACGATCC	Not available for all sequences	Not available
Hvu-miRX6	ATTTACTTGTAGAAGAAGCTA	10/11: ACAAGTAAATACGACATGGG	gb|HQ825319.1|^/^gb|AF147501.1|	ribosome protein
Hvu-miRX7	TCAGATGAGAAGGCAGATCATA	10/11: TCTCATCTGAGTTTGATCCT10/11: TCTCATCTGAAATATGATCCT	Not available for all sequences	Not available
Hvu-miRX8	TAGGAAAGTAGAGTAGGCACA	11/12: CTACTTTCCTACAAAGTTAT	Not available	Not available
Hvu-miRX9	ATTGACGACCTAGATACACGTGCA	12/13: TAGGTCGTCAATCCTACCAT	Not available	Not available
Hvu-miRX10	TCTGTAACTTAATATAAGACG	14/15: TATTAAGTTACAGAGGGAGAG	Not available	Not available
Hvu-miRX11	TATTTGCAGGTTTTAGGTCTAA	10/11: CCTGCAAATAATATAGCATGT10/11: CCTGCAAATACCTGGTTCTC	Not available for all sequences	Not available
Hvu-miRX12	AATTATTTAGGTACAGAGGGA	10/11: CTAAATAATTTAAAAATGGA	Not available	Not available
Hvu-miRX13	AATTAATATGGATCGGAGGGA	10/11: CATATTAATTTCGACAACCC	Not available	Not available
Hvu-miRX14	CCGAACTGATGGAAAGGGCTA	10/11: ATCAGTTCGGCAGAGAGCAGA	Not available	Not available
Hvu-miRX15	TTTTGGTTGCGTTGGCTAGTGCAT	10/11: GCAACCAAAAGTCCGAACTAA	CA023038	DNA-directed RNA polymerase subunit
Hvu-miRX16	TATAGTAATGATGGCTAATGGT	10/11: CATTACTATACCTTCCCTATT	gb|EF115541.1|/emb|X14108.1|/gb|M35616.1|/BLYCPPSBEF/gb|M35977.1|/BLYCPBEF/TC276030	psbE and psbF proteins,b559 apoprotein
Hvu-miRX17	TTAGGATTAGGAATAGGTGTA	10/11: CTAATCCTAAGGATTCCTTAA	Not available	Not available
Hvu-miRX18	AAATAGAATAATGATCAACGGA	10/11: TATTCTATTTCACTATTTCA10/11: TATTCTATTTTTGTTGGATC	BE437950/gb|EF115541.1|Not available for “TATTCTATTTTTGTTGGATC”	unknown protein
Hvu-miRX19	TTTGAGGGTTCTAGTCTTTGC	10/11: GTGTAGAACCCTCAAATGAG	Not available	Not available
Hvu-miRX20	ATCGTATGAACTTGAAGCAACGGT	10/11: TTCATACGATCCAACGAGTTC	Not available	Not available

Note: Sequences highlighted in red in each row are from the same target. Target cleavage sites are indicated by numbers relative to the guiding miRNAs from 5′ to 3′.

### Overall ncsRNAs in Transgenic and Non-transgenic Barley

The reads not being assigned to a miRNA were aligned with the Rfam database (allowing 2 mismatches), a comprehensive collection of ncRNA families represented by multiple sequence alignments. 664 ncRNAs were mapped in non-transgenic barley and 495 mapped in transgenic barley, of which 260 ncRNAs are specific to non-transgenic barley, while 89 are specific to transgenic barley (Table S4). Among these mapped ncRNAs, 4 are homologous to piRNAs (1 from transgenic barley and 3 from non-transgenic barley), but were all low abundant (Table S4). Considering that piRNAs have never been reported in plants, these piRNA homologs may not be bona fide piRNAs.

### rasiRNAs in Transgenic and Non-transgenic Barley

To analyse rasiRNAs, all the reads were aligned with the Triticeae repeat sequence database (TREP), which contains 1,716 transposable elements (TEs). A total of 3823 reads from transgenic barley and 11978 reads from non-transgenic barley mapped to the TREP database. The mapped reads accounted for 0.37% of the total reads in transgenic barley and 0.8% in non-transgenic barley. These percentages are low, considering that about 80% of the barley genome is composed of repetitive sequences primarily consisting of TEs. Altogether, 433 TE-derived sRNAs in transgenic barley and 784 in non-transgenic barley were mapped, of which 132 were significantly down-regulated, while 2 were up-regulated, in transgenic barley (|log_2_| >1) (Table S5). Notably, all the TE-derived sRNAs in transgenic barley are present in non-transgenic barley.

Among the mapped TE-derived sRNAs in transgenic barley, 186 belonged to retrotransposons (Class I TEs), while 169 to DNA transposons (Class II TEs), accounting for 43% and 39%, respectively (Table S6). The unclassified TE-derived sRNAs accounted for 18% of the total mapped TE-derived sRNAs (Table S6). In non-transgenic barley, 387 belonged to retrotransposons, 330 to DNA transposons and 67 were unclassified, accounting for 49.4%, 42.1% and 8.5%, respectively (Table S6). The precent of each TE class in one group appeared to be similar in another group, suggesting that the regulation of TE-derived sRNAs in transgenic barley may not depend on the TE type, but on the expression levels. Similar results were observed when the reads were aligned to other repetitive databases such as Repbase (Table S7), TIGR rice repeat database (Table S8), and TIGR barley repeat database (Table S9).

### natsiRNAs in Transgenic and Non-transgenic Barley

natsiRNAs are another type of siRNAs generated from double-stranded RNAs. To identify natsiRNAs, all the reads were first mapped to the reverse strand of barley genes available in the databases. The “anti-sense” reads were then mapped to the forward strand of all other libraries (tRNA, genes, repeats, etc.). The results showed that 14,018 natsiRNA clusters existed in non-transgenic barley, while 8,297 were present in transgenic barley. Among them 3564 were common between the two groups, of which about half were significantly up-regulated in transgenic barley (Table S10). A lot of natsiRNA originated from TEs, suggesting that TEs can generate both sense and antisense sRNAs.

Some read clusters were generated from different transcripts and each targeted more than two genes. On the other hand, the same genes can be targeted by different read clusters. The redundancy of targeted genes accounted for 82.6% of the total mapped genes in non-transgenic barley and 84.3% in transgenic barley. Despite so, about 2/3 of the mapped genes were only targeted by single reads.

The most abundant natsiRNA in the two groups was derived from a ribosomal RNA, while the second most abundant natsiRNA in the two groups originated from P450 mRNA. Both natsiRNAs were more abundant in non-transgenic barley than in transgenic barley. Interestingly, both natsiRNAs had their sense counterparts in the groups. It is not clear which type of sRNA of these two natsiRNAs is really functional in vivo. The abundance of each of the other natsiRNAs was not consistent between the two groups, suggesting the expression of natsiRNAs was affected by the TF.

### tsRNAs in Non-transgenic and Transgenic Barley

tsRNAs are a novel class of sRNAs presented in both plants and animals [12], [25]. 55 tsRNAs were identified in non-transgenic barley, while 54 were identified in transgenic barley (Table S11). The extra tsRNA in non-transgenic barley was derived from tRNA-LeuCAG. Of the identified tsRNAs in the two groups, tsRNA derived from tRNA-Gly(TCC) was the most abundant, accounting for 83.1% of the total tRNA-derived reads in non-transgenic barley and 57.9% in transgenic barley, followed by tRNA-Ala(AGC) derived sRNA, accounting for 8.4% in non-transgenic barley and 14.1% in transgenic barley (Table S11). Most identified tsRNAs were differentially expressed between the two groups, of which 6 tsRNAs were significantly up-regulated (log2> = 1), while 20 tsRNAs were significantly down-regulated (log2< = −1), in transgenic barley (Table S11). Distinctly, the most abundant tRNA-Gly(TCC)-derived sRNA in non-transgenic barley was 4 times higher in read count than in transgenic barley (Table S11). The varied expression levels may signify that the coordination may be adopted for tsRNAs in responsible for the over-expressed *TaDREB3* in the cells.

### sRNAs Derived from Transgenic *TaDREB3* and its Downstream Regulated Genes

**Figure 4:**
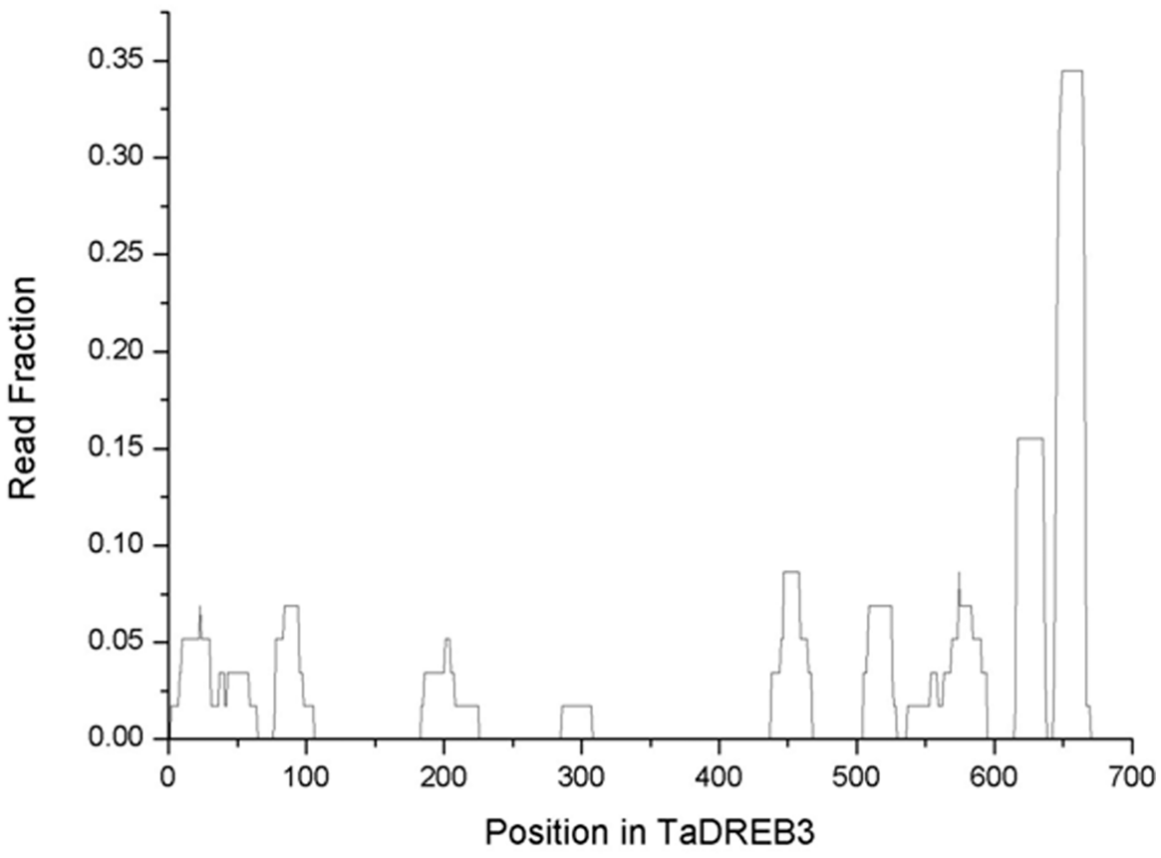
Distribution of small RNAs on TaDREB3. Position and read fraction are depicted.

To determine if there were reads derived from *TaDREB3*, the remaining reads were aligned to the *TaDREB3* sequence. 57 reads from transgenic barley were perfectly matched to *TaDREB3* (Table S12). In contrast, none of the reads from non-transgenic barley were matched despite the existence of homologous genes in the plant (our unpublished data), thereby confirming that the matched reads were derived from the *TaDREB3* transcripts. Most of the matched reads localised at the 3′ region of the gene (Figure 4). Only a few reads, or no read, were mapped to other regions (Figure 4). Intriguingly, a couple of reads from transgenic barley, but not from non-transgenic barley, were perfectly matched to the antisense strand of the *TaDREB3* transcripts (Table S12) and the matched regions right overlapped with the mapped sense-strand regions. These co-incidences may indicate the existence of a feedback loop in transgenic barley, in which the transcribed *TaDREB3* initiated the generation of sense sRNAs through normal degradation process, then the generated sense sRNAs function as siRNAs to cleave double-stranded structures of the *TaDREB3* transcripts to produce anti-sense sRNAs, and the resulting antisense sRNAs cleave *TaDREB3* to release sense sRNAs.

**Figure 5:**
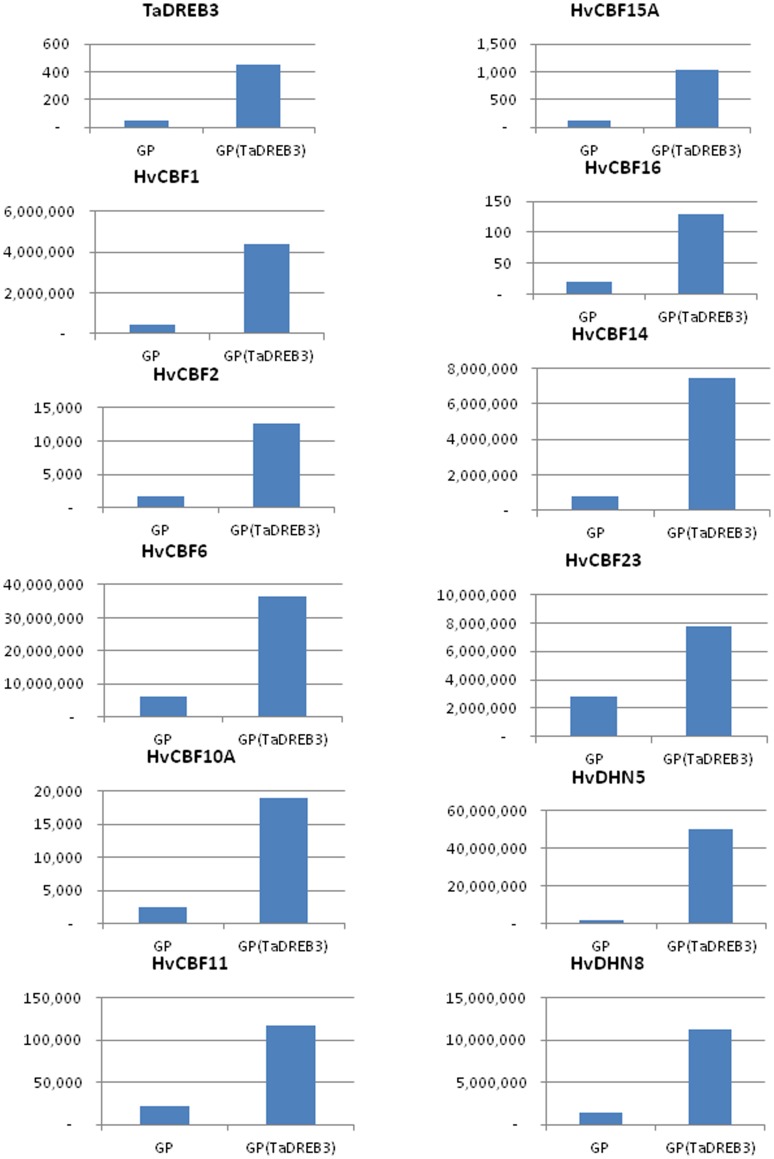
Expression levels of TaDREB3 and its regulated downstream genes measured by qRT-PCR in non-transgenic barley and transgenic barley. The bar graph shows fold enhancement of the transcripts of the TaDREB3 and its regulated downstream genes relative to the transcripts of the four control genes as described in the text. All the TaDREB3 regulated downstream genes in transgenic barley were expressed at levels higher than the same genes in non-transgenic barley.

sRNAs derived from *TaDREB3*-regulated genes such as *HvCBF15, HvCBF16, HvCBF10B, HvCBF23, HvCor14B, HvDHN5, HvDHN8, HvDHN9* and *HvDHN10* were also detected in both groups. However, the mapped read number and read count were more in non-transgenic barley than in transgenic barley. In addition, the *HvDHN8* gene was only mapped by the reads from non-transgenic barley, but not from transgenic barley. Curiously, antisense reads to these genes were also detected in the two groups, but the mapped read number and read count were again more in non-transgenic barley than in transgenic barley (Table S12). qRT-PCR showed that all of the above genes were expressed at higher levels in transgenic barley than in non-transgenic barley (Figure 5), confirming that these genes were up-regulated by *TaDREB3*. *HvCBF16* was expressed at the lowest level in both groups (Figure 5). Correspondingly, the detected read number and read count reverse complementary to *HvCBF16* were the highest among all the genes (Table S12), suggesting that the expression of *HvCBF16* might be regulated through the miRNA/siRNA pathway.

### Profile of sRNAs Derived from Chloroplast in Transgenic and Non-transgenic Barley

**Figure 6:**
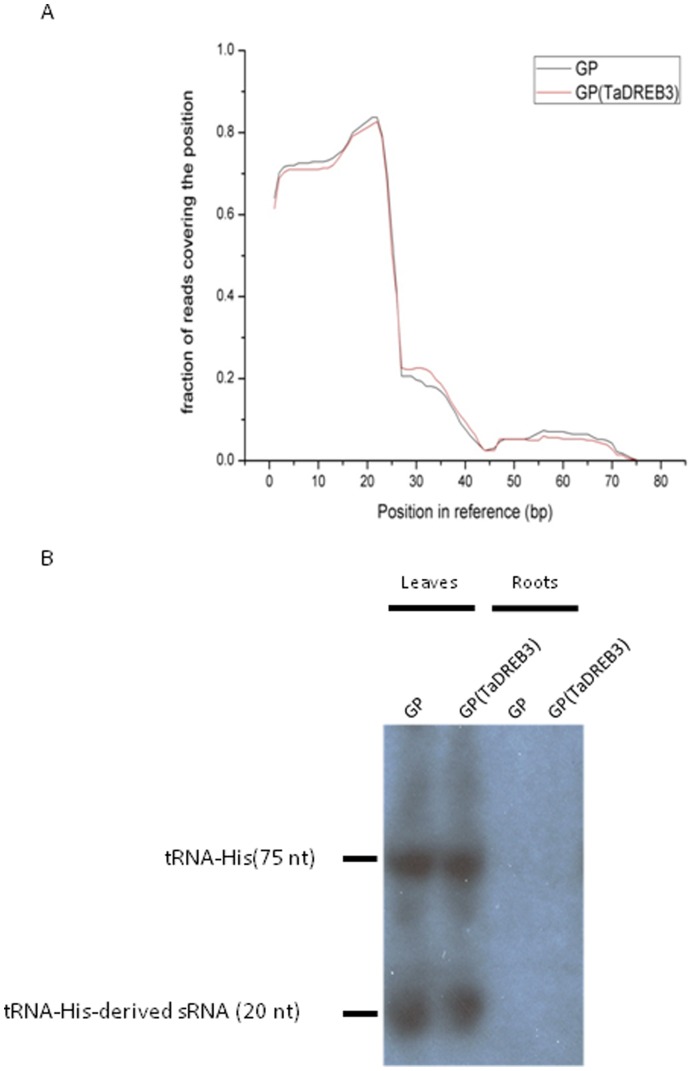
Expression of tRNA-His(GTG)-derived sRNAs in barley. (A) Fraction of unique reads that cover each position of the tRNA-His(GTG) gene. (B) Northern blot hybridization of sRNAs derived from tRNA-His(GTG). Note that we depict the distribution of unique reads here in order to visualize the mappings to other positions. The read count is completely dominated by few reads that map to the 5′ end of the gene which would make it impossible to see the other mappings. The transcripts of tRNA-His(GTG) and the tRNA-His(GTG)-derived sRNAs were hardly detected in the root tissues.

Next, we further analysed csRNAs in transgenic and non-transgenic barley. We found that the chloroplast reads were distributed across almost the whole chloroplast genome regions in both orientations (Table S13), suggesting that a normal degradation process is dominant. However, the reads mapped to the regions encoding tRNAs, rRNAs, PetB and PsbA appeared to be more than those mapped to intragenic, intergenic or other gene regions. In addition, the predominant sizes mapped to tRNAs and rRNAs in both groups were 20 nt and 19 nt, while there was no predominant size mapped to other RNAs or regions, suggesting that the degradation process is discriminating and that tsRNAs or rRNA-derived sRNAs (rsRNAs) might be processed in a special way. A few chloroplast reads from the two groups were mapped to several TEs (Table S14). Surprisingly, a tRNA-His(GTG) derived sRNA from the chloroplast genome, which matched to the 5′ end of the tRNA-His (GTG) gene, was the most abundant of all the reads sequenced (Table S15). Because no other abundant reads were mapped to the other regions of the tRNA-His (GTG) gene (Table S15, Figure 6A), we speculate that this most abundant tsRNA may be generated under a special mechanism. Interestingly, this tsRNA was only present in the leaves, but not roots (Figure 6B), which further supports its chloroplast origin because plastids in roots do not develop chloroplasts. Curiously, this tsRNA was found to perfectly match to the antisense strand of the 50S ribosomal gene (TC245676), which was also from the chloroplast genome. Whether the tsRNA and the gene interact in vivo is worthy of further investigation.

## Discussion

Transcriptional regulation in eukaryotes is known to occur through the coordinated action of multiple TFs with other transcriptional regulators such as miRNAs and other classes of sRNAs. To gain insights into this cooperative network, we used next-generation sequencing technology to deeply sequence sRNAs in transgenic barley over-expressing the *TaDREB3* TF and in non-transgenic control barley. Comparison of these sRNA expression profiles between the two groups revealed that the TF strongly affects the sRNA expression.

24 miRNAs were absent in transgenic barley (Table S1), suggesting that the expression of these miRNAs might be suppressed by *TaDREB3*. On the other hand, 20 miRNAs were transgenic barley specific (Table S1) implicating that these miRNAs might be activated by *TaDREB3*. Combined together, these results indicate that *TaDREB3* affects the miRNA expression in multiways. It is to note that most of these group-specific miRNAs are low copies because when threshold > = 10 is applied, only one miRNA is different between the two groups (Supplemental Table 1). This highlights the possibility that the low abundant group-specific miRNAs may be sequenced by chance in one sample but not in the other and thus not be truly group specific. Why some miRNAs were activated, while others were suppressed, by *TaDREB3* is not clear, but likely to be associated with the function of *TaDREB3*. It is conjectured that most of these miRNAs may not be directly regulated by *TaDREB3* because the binding site for *TaDREB3* was not found in the upstream region of most miRNA genes that were regulated by *TaDREB3*. Instead, these miRNAs might be regulated through *TaDREB3*-related TFs or by means of regulating the miRNA host genes (in case of intronic miRNAs). In support of this, the predicted putative targets of miRNAs were not found to be the targets of *TaDREB3* (Table S3).

Differentially expressed miRNAs were observed between the two groups. The most abundant miR156 was over two-fold up-regulated in transgenic barley. Coincidently, miR156 is induced by drought [26] and *TaDREB3* is specially functional in drought tolerance and induced by drought [14]. This suggests that the regulation of miR156 in vivo may be true and miR156 is a good candidate for drought tolerance and for further functional analysis of the impact of *TaDREB3* on the expression of miRNAs. Previous studies showed that miR156 targets SQUAMOSA promoter binding protein-like (SPL) TFs, which control flowering time, phase change, leaf initiation rate and positively regulate miR172 [27]–[28]. Therefore, miR156 suppresses the expression of miR172. Correspondingly, miR172 was down-regulated in transgenic barley (Table S1). Intriguingly, miR172 targets the AP2 genes, which can also control the timing of flowering [29] and enhance drought tolerance [30]. These data indicate that differentially expressed miRNAs are connected to each other in complex networks in order to coordinate with the function of *TaDREB3*. It is to note that miR172 can in turn positively regulate the SPL TFs, but not miR156 [28].

Analysis of the putative miRNA sequences showed that uracil and adenine were the two predominant nt at the 5′ ends, accounting for 45% each, while adenine was a predominant nt at the 3′ ends, accounting for 55% (Table 8). The preference of these nt at the 5′ and 3′ ends is in agreement with previous findings in rice and Arabidopsis [31]. However, in the miRBase, the most dominant uracil at the 5′ ends accounts for 76% in Arabidopsis and 58% in rice, followed by adenine, accounting for 12% in Arabidopsis and 11% in rice. The 5′ terminal nt of miRNAs has been implied to be an important recognition signal for the argonaute (AGO) proteins [32], which are central to the function of an active RNA-induced silencing complex (RISC) containing Dicer and associated proteins. The 5′ terminal uracil is preferentially recognised by AGO1, while the 5′ terminal adenine is preferentially recognised by AGO2 [32]. The size of miRNAs also has an effect on the selection of AGOs. 24-nt miRNAs are preferentially recognised by AGO4, while the other sizes of miRNAs as well as the 24-nt miRNAs with C at the 5′ end are preferentially recognised by AGO5 [32].

Alignment of sRNA sequences with the *TaDREB3* gene revealed that some sRNA sequences were derived from *TaDREB3*. We observed that (i) these sRNA sequences were only detected in transgenic barley, but not in non-transgenic barley containing the *TaDREB3*-homologous gene; (ii) the *TaDREB3*-derived sRNA sequences were concentrated on some regions rather across the whole gene; and (iii) sRNA sequences reversely complementary to the *TaDREB3* gene were only detected in transgenic barley and some overlapped forward *TaDREB3*-derived sRNA sequences. These observations suggest that *TaDREB3* may be degraded at least partially via the miRNA/siRNA pathway. If this hypothesis is true, it could explain why so many sRNA sequences are different between the two groups and would be the first direct evidence for the feedback loop model between TFs and sRNAs in plants: *TaDREB3* activates the expression of sRNAs and the activated sRNAs in turn regulate the expression of *TaDREB3*. Nevertheless, this model needs to be confirmed experimentally.

A number of sRNA sequences were mapped to the *TaDREB3*-regulated genes, but differ in number and read count between the two groups. In addition, only the reads from non-transgenic barley, but not transgenic barley, were mapped to the *TaDREB3*-regulated *HvDHN8* gene (Table 12). On the other hand, both sense and antisense sRNAs derived from the *TaDREB3*-regulated genes existed (Table S12), some of which were mapped to the same regions of these genes (data not shown). These lead us to speculate that *TaDREB3* and sRNAs may both be involved in the regulation of the *TaDREB3*-regulated genes and that the processing of these genes into sRNA fragments may be subject to the miRNA/siRNA pathway. However, following a normal degradation pathway is also possible because the reads were distributed across the whole regions of these genes. In addition, it cannot be ruled out that other TFs may also participate in the regulation of the DREB3-regulated genes. Taken together, the regulation of these DREB3-regulated genes may be a complex process.

To our surprise, a tRNA-His(GTG)-derived sRNA accounts for approximately 1/3 of the total sRNAs sequenced, thereby making it the most abundant sRNA and making tsRNAs the most abundant sRNAs in the two groups. This phenomenon had never been reported before in any other organism. In wheat, the same tsRNA only exists in a small number (our unpublished data) despite that wheat and barley are genetically close to each other. tRNAs not only have a role in translation, but also function in other cellular processes such as reverse transcription, porphyrin biosynthesis and others [33]. We expect that tsRNAs, especially the most abundant tsRNA, could have the related functions as above. This might be true because a recent study showed that the silencing of a tsRNA (tRF-1001) in human prostate cancer cells through a siRNA strategy decreases the cell viability [11]. On the other hand, it is also possible that tsRNAs, especially the most abundant tsRNA, may play a different role in barley from their counterparts in other species. Consistent with this possibility, we found that tsRNAs including the tRNA-His(GTG)-derived sRNAs were not randomly generated in barley (Table S14). In addition, we found that the most abundant tsRNA mapped to the reverse complementary strand of a gene (TC245676) involved in sRNA binding (GO:0003723), biological processes (GO:0006412), ribosome (GO:0005840 and GO:0003735) and plastid (GO:0009536). Due to the lack of bioinformatics, we did not pursue the identification of tsRNA targets.

A large number of sRNAs were mapped to the chloroplast genome (or chloroplast-derived sequences inserted at the nuclear genome) (Table S16). Similar situation has been described in rice [17] and Arabidopsis [34]–[35]. This indicates that csRNAs may have conserved functions among different species. However, some csRNAs might be by-products of chloroplast RNA processing, which may not be functionally relevant. Intriguingly, many barley csRNAs were mapped to intergenic regions between the psbB, psbT, psbH, petB and petD genes (our unpublished data). In particular, one of these regions was previously identified to contain a sRNA binding site for the cleavage of the polycistron [36]. This suggests that at least part of these csRNAs may result from the miRNA/siRNA pathway. This would then reflect a more ancient application of sRNA-directed transcript processing. Exploration of the enzymatic machinery would help determine sRNA processing in the chloroplasts.

Previous studies showed that TFs activate TEs. However, we found that the TE-derived sRNAs in transgenic barley were less in both number and read count than those in non-transgenic barley. This indicates that *TaDREB3* may not be responsible for the activation of TEs. On the other hand, the detected transgenic barley-specific TE-derived sRNAs suggest that *TaDREB3* is responsible for the activation of some TEs. Taken together, these indicate that *TaDREB3* may have a dual role in the regulation of the TE-derived sRNAs. It is likely that *TaDREB3* may regulate the TE-derived sRNAs through other TFs or siRNAs, the latter of which specifically function in silencing TEs. If it is true, because part of siRNAs may be generated from endogenous double-stranded RNAs (dsRNAs), which are processed from the inverted repeats, then a possible feedback loop may exist in transgenic barley, in which the TF transcribes TEs, and siRNAs generated from the transcribed TEs repress the TF, thereby reducing the number and abundance of TEs. It is to note that siRNAs can be generated from other classes of TEs and a complicated network may be involved in the generation. This would make it difficult for siRNAs to function consistently in silencing TEs. Any subtle change in the network could change sRNA expression patterns and functions significantly.

We have mentioned above that the miRNA/siRNA pathway may be adoptedd by different classes of sRNAs. This would raise the possibility that these sRNAs might compete with each other for Argonautes for their biogenesis and functions. In mouse and yeast loss of Dicer has been shown to increase one class of sRNAs, but decrease another [37]–[38], thereby confirming the existence of competition. However, such competition may be intricate and vary with the physiologic state of the cell. It may be inferred that each class of sRNAs has its own accessories, which allow avoiding using Argonautes for their biogenesis at the same time in the same cells. This means that while one class of sRNAs is generated, another class of sRNAs stands or decays. This strategy could be adopted by sRNAs within the same classes for their biogenesis. This could partly explain why different abundant sRNAs exist in the same cells. However, we cannot rule out the involvement of other unknown mechanisms in the generation of these sRNAs.

In summary, we present a comprehensive analysis of the sRNA components in barley including miRNAs, rasiRNAs, natsiRNAs, tsRNA, csRNAs and other non-coding sRNAs. Because the two groups only differ in *TaDREB3*, any difference in the expression of sRNAs between the two groups should be related to *TaDREB3*. Thus, our data increases our knowledge of the relationship of sRNA expression and *TaDREB3*. However, fundamental studies to assign the mechanisms of biogenesis and biological functions of the TF-related sRNAs are needed.

## Methods

### Barley Cultivar

The transgenic barley (*H. vulgare* cv. Golden Promise) with*TaDREB3* has been described previously [14]. The presence of the transgene has been confirmed by Southern and Northern Blot Hybridization [14]. For water use efficiency (WUE) analysis, transgenic plants of the T3 generation were grown in 6-inch pots in a growth room with a 16-hour day length at day/night temperatures of 24°C/16°C for four weeks and water was then withheld for seven days. WUE was calculated in grams of dry shoot biomass per ml of water used. Leaves of watered transgenic and non-transgenic plants were harvested after a four-week growth for sRNA isolation.

### sRNA Isolation and Sequencing

sRNAs were isolated using Purelink miRNA Isolation Kit (Invitrogen, Carlsbad, CA, USA) from total RNA extracted with Trizol reagent (Invitrogen, Carlsbad, CA, USA) from leaf material pooled from 3 individuals from the same transgenic or non-transgenic line. After quality assessment by Bioanalyzer, sRNA preparations were adjusted to the same concentration and electrophoresized on a 15% polyacrylamide (30∶0.8) gel containing 7 M urea in TBE buffer (45 mM Tris-borate, pH 8.0, and 1.0 mM EDTA). 18–30 nt sRNAs were excised from the gel and recovered in 0.3 M NaCl. The recovered 18–30 nt sRNAs were then ligated with both 5′ adaptor (5′-GUUCAGAGUUCUACAGUCCGACGAUC-3′) and 3′ adaptor (5′- pUCGUAUGCCGUCUUCUGCUUGUidT-3′, p and idT represent phosphate and inverted deoxythymidine, respectively) using T4 RNA ligase and subject to RT-PCR. After purification from the gel as above, RT-PCR products were adjusted to the same concentration and loaded into the same flow cell for sequencing on the Illumina Genome Analyser.

### Construction of Barley Degradome Library

The degradome library was constructed as described before [39]. Briefly, poly(A) RNA, extracted from total RNA from Golden Promise barley with the Oligotex kit (Qiagen), was ligated with a 5′ RNA adaptor containing a *Mme*I restriction site using T4 RNA ligase (Invitrogen), followed by reverse transcription, second-strand synthesis, *Mme*I digestion, ligation of a 3′ dsDNA adaptor, gel-purification, and PCR amplification. Amplified PCR products were sequenced with the Illumina HiSeq platform.

### Trimming of sRNA Sequences and Bioinformatics Analysis

The sequence reads obtained were processed as follows: after removing poly A, T, C, G and/or N containing reads, all reads were grouped which had the same read sequences termed “unique reads”. Next the 3′ adapter sequence was removed using the following parameters: (i) at least 10 nt of the adapter needed to be detected, (ii) 1 mismatch was allowed between the adapter sequence and the read, (iii) the search for the adapter sequence was started at the 18th base of the read. After the removal of all untrimmed reads (no adapter was detected), the reads were regrouped in order to calculate the read-count of unique read sequences. All trimmed reads were compared to the complete barley chloroplast genome sequence without mismatches and then separated into non-chloroplast reads and chloroplast reads. To profile the expression of known miRNAs and other transcribed sequences, we used the miRanalyzer standalone version [23]. This version was launched allowing 0 mismatches for all libraries with the exception of RFam (2 mismatches allowed). For all other parameters the default values were used. Furthermore, after mapping the reads to a given library we set miRanalyzer to remove the mapped reads so they could not map to any other libraries. To profile the expression of known miRNAs we generated a non-redundant reference library of the miRBase release 17. In order to obtain a non-redundant library we grouped together all mature miRNAs, which have the same sequence. MiRanalyzer allows for the customization of additional libraries to which the reads should be mapped. To identify sRNAs derived from repeats such as (retro)transposons we aligned the reads with a minimum length of 20 bp to the following libraries: (i) barley and rice libraries from the TIGR repeat database [40], (ii) complete TREP repeats [41] and (iii) all RepBase repeats [42]. To identify sRNAs corresponding to ribosomal RNAs (rRNAs), transfer RNAs (tRNAs), small nuclear RNAs (snRNAs), small nucleolar RNAs (snoRNAs), spliceosome RNAs (U1–U6) and other annotated ncRNAs, we compared the reads with sequences extracted from the Rfam database containing various ncRNA families. To analyse antisense sRNAs (siRNAs), we first removed the reads mapped to known miRNAs and then aligned the remaining reads to the reverse strand of all barley genes available in the databases. The reads hitting the same genes were grouped into ‘read clusters’ based on their sequence similarity. The “anti-sense” reads were then mapped to the forward strand of all other libraries (tRNA, genes, repeats, etc.). Because a gene could be hit by different reads (different length variants or from different genome elements), we clustered similar reads together (reads that are perfect inclusions of each other). For each read cluster an output line was generated with the gene, the reads, the read count, and the sequence element where the read might come from. Mapping the reads to *TaDREB3* and its regulated downstream genes were performed using the same method as above.

### Prediction of Novel miRNAs and their Target Genes

Given the absence of a genome assembly we tried to identify miRNAs by means of a homology based approach. After analysing the known miRNAs of barley, we mapped the remaining reads to a non-redundant library of all known miRNAs from the miRBase allowing 0 mismatches. MiRNAs tend to be conserved and therefore many of the miRNAs identified in related species will also be present in barley although they have not yet been detected.

For detecting putatively novel miRNAs, all the remaining reads were mapped to barley (allowing 1 mismatch), wheat, *Brachypodium distachyon* (L.) or rice (*Oryza sativa* L.) (allowing up to 2 mismatches) EST or genomic sequences in order to search for the presence of hairpin structures, a key characteristic of miRNAs. The putative pre-miRNA secondary structures and the mean free energy were predicted by MFOLD [43]. Only the precursors with free energy lower than −18 kcal/mol were maintained for experimental validation. Target genes were predicted using psRNATarget [44], a plant sRNA target analysis server (http://plantgrn.noble.org/psRNATarget).

### Northern Blot Hybridization

50 µg of total RNA was separated on a 15% polyacrylamide gel containing 7 M urea and then transferred to Hybond-N membrane (Amersham Bioscience) using 20×SSC. The membrane was hybridized with 32P labelled DNA oligonucleotide probe reverse complementary to predicted miRNA sequence, made with γ-32P-ATP using T4 polynucleotide kinase (New England Biolabs, Beverly, MA). U6 served as a loading control and its probe was made in the same way as above. Prehybridization and hybridization were both performed at 42°C in ULTRAhyb Ultrasensitive Hybridization Buffer (Ambion). Washing was also performed at 42°C using 2×SSC, 0.1% SDS, twice followed by 1×SSC, 0.1% SDS once. The membrane was either imaged using a Phosphorimager (Typhoon Trio, Amerisham Bioscience) or exposed to an X-ray film.

### RT-PCR and Quantitative Real-time PCR

RT-PCR was performed as described [13] and quantitative real-time PCR (qRT-PCR) with primers was carried out according to Morran et al. [14]. Briefly, leaf tissues were collected from barley plants grown under well watered and drought conditions. Total RNA was extracted from the tissues using the Trizol reagent. Reactions were performed in an RG 6000 Rotor-Gene real-time thermal cycler (Corbett Research): 3 minutes at 95°C followed by 45 cycles of 1 second (s) at 95°C, 1 s at 55°C, 30 s at 72°C (fluorescence reading acquired), and 15 s at 81°C. The transcript levels of genes and control genes encoding glyceraldehyde 3-phosphate dehydrogenase, heat shock protein 70, cyclophilin, and α-tubulin were normalised as described [45].

## Supporting Information

Table S1 **Conserved miRNAs mapped by reads in non-transgenic barley (GP) and transgenic barley (GP(TaDREB3)).** The log2-ratio indicates the differential expression. Sheet 1 shows conserved miRNAs mapped by the reads with threshold > = 10. Sheet 2 shows conserved miRNAs mapped by the reads with threshold > = 1.
(XLSX)

Table S2 **Primers used in RT-PCR of putative novel miRNAs.**
(XLSX)

Table S3 **Predicted targets of putative novel miRNAs.**
(XLSX)

Table S4 **Detected ncRNA families in the rfam database by reads (allowing 2 mismatches) in non-transgenic barley (GP) and transgenic barley (GP(TaDREB3)).** The log2-ratio indicates the differential expression. Sheet 1 shows detected ncRNA families by the reads with threshold > = 1. Sheet 2 shows detected ncRNA families by the reads with threshold > = 10.
(XLSX)

Table S5 **Detected TEs in the TREP database by reads (allowing 0 mismatch) in non-transgenic barley (GP) and transgenic barley (GP(TaDREB3)).** The log2-ratio indicates the differential expression. Sheet 1 shows detected TEs by the reads with threshold > = 1. Sheet 2 shows detected TEs by the reads with threshold > = 10.
(XLSX)

Table S6 **Summary of the mapped TEs by the reads in non-transgenic barley (GP) and transgenic barley (GP(TaDREB3)).** The log2-ratio indicates the differential expression.
(XLSX)

Table S7 **Detected repeats in the RepBase database by reads (allowing 0 mismatch) in non-transgenic barley (GP) and transgenic barley (GP(TaDREB3)).** The log2-ratio indicates the differential expression. Sheet 1 shows repeats detected by the reads with threshold > = 10. Sheet 2 shows summary of the repeats mapped by the reads in non-transgenic barley (GP) and transgenic barley (GP(TaDREB3)).
(XLSX)

Table S8 **Detected repeats in the TIGR rice database by reads (allowing 0 mismatch and threshold > = 10) in non-transgenic barley (GP) and transgenic barley (GP(TaDREB3)).** The log2-ratio indicates the differential expression.
(XLSX)

Table S9 **Detected repeats in the TIGR barley database by reads (allowing 0 mismatch and threshold > = 10) in non-transgenic barley (GP) and transgenic barley (GP(TaDREB3)).** The log2-ratio indicates the differential expression.
(XLSX)

Table S10 **Detected antisense small RNAs by reads (allowing 0 mismatch and threshold > = 1) in non-transgenic barley (GP) and transgenic barley (GP(TaDREB3)).** Sheet 1 shows antisense small RNAs detected by the reads (allowing 0 mismatch and threshold > = 1) in GP. Sheet 2 shows antisense small RNAs detected by the reads (allowing 0 mismatch and threshold > = 1) in GP(TaDREB3).
(XLSX)

Table S11 **Detected tRNA-derived small RNAs (tsRNAs) by the reads (allowing 0 mismatch) in non-transgenic barley (GP) and transgenic barley (GP(TaDREB3)).** The log2-ratio indicates the differential expression. Sheet 1 shows tsRNAs detected by the reads with threshold > = 1. Sheet 2 shows tsRNAs detected by the reads with threshold > = 10.
(XLSX)

Table S12 **Small RNAs derived from TaDREB3 and its regulated 10 genes in non-transgenic barley (GP) and transgenic barley (GP(TaDREB3)).** The log2-ratio indicates the differential expression.
(XLSX)

Table S13 **Chloroplast genome distribution of the reads (allowing 0 mismatch) in non-transgenic barley (GP) and transgenic barley (GP(TaDREB3)).** Sheet 1 shows chloroplast genome distribution of the reads in GP. Sheet 2 shows chloroplast genome distribution of the reads in GP(TaDREB3).
(XLSX)

Table S14 **TE-derived small RNAs detected in the chloroplast in non-transgenic barley (GP) and transgenic barley (GP(TaDREB3)).** The log2-ratio indicates the differential expression.
(XLSX)

Table S15 **Bowtie output of the read alignments with the tRNA-His gene in non-transgenic barley (GP) and transgenic barley (GP(TaDREB3)).** Sheet 1 shows bowtie output of the read alignments with the tRNA-His gene in GP. Sheet 2 shows bowtie output of the read alignments with the tRNA-His gene in GP(TaDREB3).
(XLSX)

Table S16 **Summary of all reads obtained, trimmed, unique or redundant, identified as various classes of small RNAs, unclassified or unassigned from the nuclear genome or from the chloroplast genome in non-transgenic barley (GP) and transgenic barley (GP(TaDREB3)).**
(XLSX)
